# Transcriptomics and Metabolomics Insights into the Dysregulation of Chondrocyte Differentiation Induced by T-2 Toxin

**DOI:** 10.3390/ijms262411858

**Published:** 2025-12-09

**Authors:** Shiqiang Cheng, Huan Liu, Xuena Yang, Li Liu, Bolun Cheng, Yan Wen, Yumeng Jia, Feng Zhang

**Affiliations:** NHC Key Laboratory of Environment and Endemic Diseases, Collaborative Innovation Center of Endemic Disease and Health Promotion for Silk Road Region, School of Public Health, Health Science Center, Xi’an Jiaotong University, Xi’an 710061, China; chengsq0701@stu.xjtu.edu.cn (S.C.);

**Keywords:** chondrocyte differentiation, Kashin-Beck disease, T-2 toxin, ATDC5 cell line, transcriptomic, metabolomic

## Abstract

T-2 toxin, a potent mycotoxin produced by *Fusarium* species, is recognized for its neurotoxic, hepatotoxic, and reproductive toxic effects. However, its impact on chondrocyte differentiation and cartilage development is not well understood. This study combines transcriptomic and metabolomic analyses to investigate the impact of T-2 toxin on ATDC5 cells during differentiation at days 3, 7, 14, and 21, following 48 h of exposure. Additionally, real-time quantitative PCR (RT-qPCR) was used to evaluate the expression levels of key chondrocyte differentiation-related genes (e.g., *Fn1*, *Sox9*, *Runx2*, *Acan*, *Col1a1*, *Col2a1*, and *Col10a1*) under T-2 toxin exposure alone or in combination with recombinant Mouse BMP2, BMP4, or both. Our results show that T-2 toxin disrupts several genes and signaling pathways crucial for chondrogenesis, including the BMP signaling pathway, extracellular matrix organization, and arachidonic acid metabolism. Significant temporal alterations in *Bmp2*, *Bmp4*, and other key genes were observed, disrupting chondrocyte differentiation. Notably, these changes were mitigated by BMP2 and BMP4 co-treatment, highlighting their protective effects. These findings provide insights into the molecular mechanisms underlying T-2 toxin-induced disruptions in chondrocyte differentiation and support the potential use of BMP signaling pathways as the therapeutic strategies to counteract the detrimental effect of T-2 toxin exposure.

## 1. Introduction

T-2 toxin, a prominent member of the trichothecene family, is frequently detected in various cereal grains, including wheat, barley, oats, and maize [1]. Recent studies have also identified its presence in nuts and dried fruits, with a detection rate of 47.6% among 233 samples in China [2]. Produced by several *Fusarium* species, such as *F. sporotrichioides*, *F. poae*, and *F. acuminatum* [3,4], T-2 toxin exhibits stable physical and chemical properties, making its removal from food and feed particularly challenging [5]. Consequently, it has become a pervasive contaminant in both human and animal food supplies. Due to its potent toxicity, T-2 toxin is known to induce various adverse health effects, including neurotoxicity, cardiotoxicity, hepatotoxicity, and reproductive toxicity, as reported in previous studies [5,6,7,8].

In addition to its established toxic effects, T-2 toxin has been identified as a significant risk factor for Kashin–Beck disease (KBD), with extensive research highlighting its detrimental impact on cartilage development [9,10,11]. KBD is an endemic chronic osteochondral disease that primarily affects the epiphyseal growth plate and articular cartilage [12]. As the growth plate cartilage serves as the growth center of bone, the developmental deformities observed in KBD patients are likely attributed to impaired chondrocyte differentiation and disrupted endochondral ossification [13]. A previous review emphasized that various signaling pathways, including BMP and TGF-β, cooperate to shape skeletal morphology during the cartilage primordium stage and regulate bone growth and maturation within the growth plates [14]. However, despite advancements in understanding the effects of T-2 toxin on chondrocyte differentiation and bone development, a comprehensive study systematically investigating the transcriptomic and metabolomic changes in chondrocytes at different differentiation stages following T-2 toxin exposure remains lacking.

Multi-omics analysis techniques are increasingly employed to uncover potential biomarkers and gain insights into disease-related pathogenesis and mechanisms [15,16]. Unlike single-omics data, multi-omics approaches provide a more comprehensive view of the underlying biological processes, capturing both causative changes and reactive responses that can be further validated through detailed molecular studies. The ATDC5 cell line, derived from mouse teratocarcinoma cells, is widely recognized as a chondrogenic model due to its well-characterized differentiation stages that closely resemble chondrocyte development [17]. When induced with 1% ITS (Insulin, Transferrin, Selenium), ATDC5 cells exhibit distinct stages of chondrogenesis: the resting stage at day 7, the proliferation stage at day 14, and the hypertrophic stage at day 21 [18,19].

In this study, to comprehensively understand the molecular mechanisms driving T-2 toxin-induced chondrocyte differentiation dysfunction and its link to KBD, we conducted an integrated transcriptomic and metabolomic analysis of ATDC5 cells exposed to T-2 toxin at days 3, 7, 14, and 21, which will offer valuable insights into the molecular mechanisms underlying chondrocyte dysfunction and its potential contribution to KBD pathogenesis.

## 2. Results

### 2.1. Transcriptomic Alterations of ATDC5 Cell Lines Induced by T-2 Toxin During Different Differential Stage

**Figure 1:**
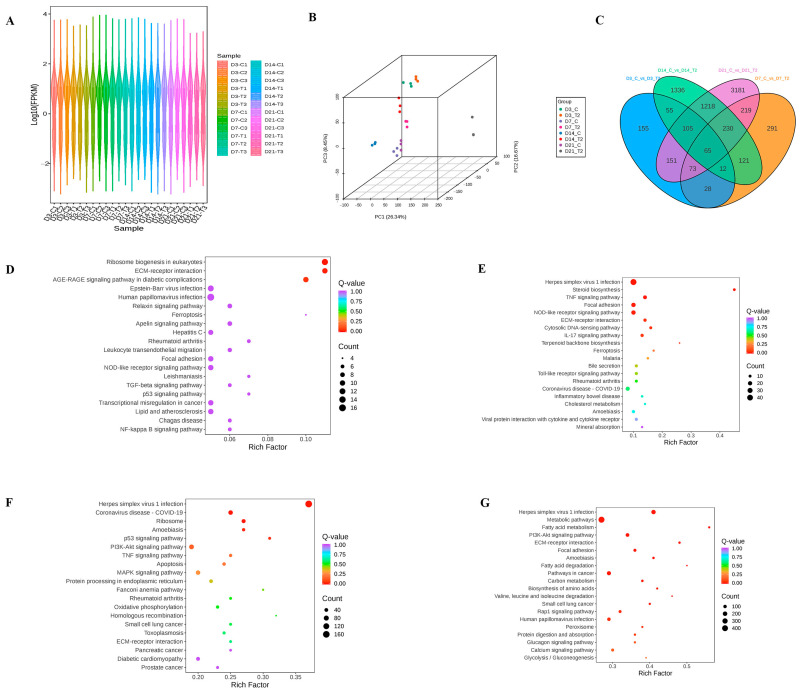
Gene expression analysis of ATDC5 chondrocyte differentiation and T-2 toxin treatment across multiple time points. (**A**) Violin plot showing the distribution of gene expression levels (FPKM) across all samples at each time point (day 3, 7, 14, 21), highlighting the overall variation between the treatment and control groups; (**B**) 3D PCA plot illustrating the clustering of samples according to T-2 toxin treatment, with distinct separation between intervention and control groups across the four time points; (**C**) Venn diagram showing the 65 common differentially expressed genes (DEGs) identified across all time points, including Col1a2 and Col2a1; (**D**–**G**) KEGG pathway enrichment analysis results of the differentially expressed genes between T-2 toxin-treated and control ATDC5 cells at day 3, 7, 14, 21.

The violin plot illustrates the distribution of gene expression levels (FPKM) across all samples at each time point (Figure 1A), and the 3D PCA plot reveals distinct clustering of samples according to T-2 toxin treatment (Figure 1B). It highlights the differences and similarities between intervention and control groups across the four time points. Among those DEG, we found 65 common genes during the overall differential process, such as *Col1a2*, *Col2a1* (Figure 1C). During the differentiation of chondrocyte model in vitro intervened by T-2 toxin for 48 h, we identified a series DEGs at different time point compared with normal controls, respectively (Supplementary Table S1). The common DEGs identified during ATDC5 cell differentiation after intervention with T-2 toxin are summarized in Supplementary Table S4.

Across all time points, the differential gene enrichment analysis consistently highlighted key GO terms and Supplementary Table S2) and KEGG pathways (Figure 1D–G and Supplementary Table S3) related to cartilage development and bone formation processes. Commonly enriched GO terms included collagen metabolic process, collagen biosynthetic process and extracellular structure organization. Additionally, at both day 3 and day 21, the differential expression analysis revealed common enrichment of the GO terms’ cellular response to BMP stimulus and regulation of BMP signaling pathway, indicating that T-2 toxin impacts BMP signaling at both early and late stages of chondrocyte differentiation. KEGG pathway analysis revealed that, regardless of the time point, T-2 toxin exposure consistently enriched pathways related to ECM-receptor interaction and focal adhesion.

### 2.2. Widely Targeted Metabolomics Analysis of ATDC5 Cell Lines Induced by T-2 Toxin During Different Differential Stage

**Figure 2:**
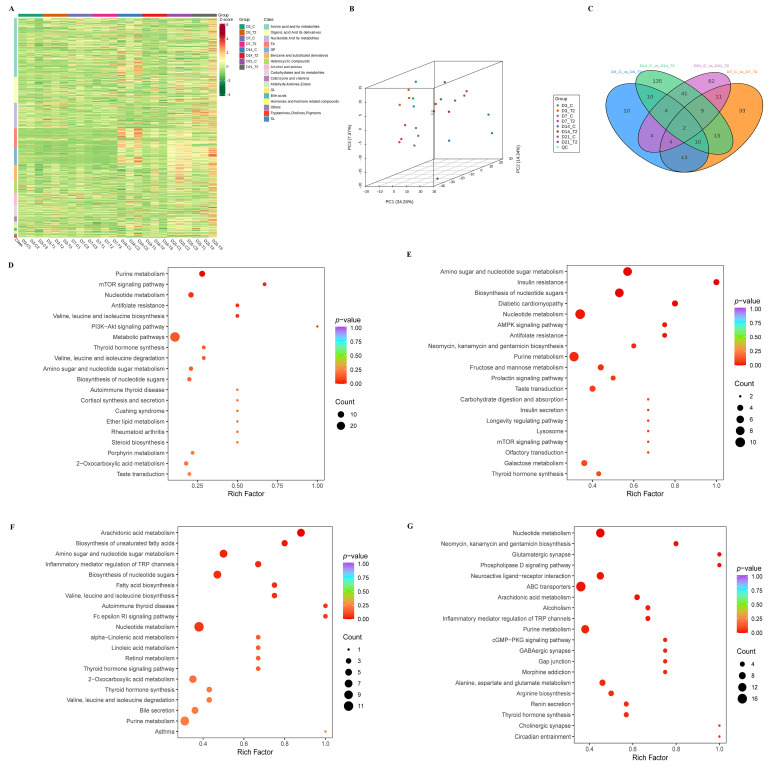
Gene Metabolomic profiling of ATDC5 chondrocyte differentiation and T-2 toxin treatment across multiple time points. (**A**) Heatmap showing the metabolic alterations associated with chondrocyte differentiation in response to T-2 toxin treatment across all samples, classified by metabolites; (**B**) PCA plot providing an overview of the metabolomic data, illustrating sample clustering and the detection of outliers, as well as the metabolic differences between T-2 toxin-treated and control groups across the four time points; (**C**) Venn diagram displaying the 2 common differential metabolites identified throughout the overall differential process, including carnitine C18:0 and Sn-glycero-3-phosphocholine; (**D**–**G**) Pathway enrichment analysis of the differential metabolites mapped to KEGG Pathway database at day 3, 7, 14, 21.

The widely targeted metabolomic profiling revealed alterations in metabolic features associated with chondrocyte differentiation in response to T-2 toxin intervention. To better capture the metabolic changes, the heatmaps were constructed to give a pictorial view using all samples classified by metabolites (Figure 2A). A PCA model was used to obtain an overview of the data, detect outliers, and evaluate the metabolomics difference among samples (Figure 2B). Among those metabolites, we found 2 common metabolites showing differences during the overall differential process, including carnitine C18:0 and Sn-glycero-3-phosphocholine (Figure 2C). A total of 38 upregulated and 19 downregulated metabolites were identified on day 3, 68 upregulated and 27 downregulated metabolites on day 7, 25 upregulated and 199 downregulated metabolites on day 14, and 122 upregulated and 35 downregulated metabolites on day 21, respectively. The difference metabolites in each time point are summarized in Supplementary Table S5. Common differential metabolites identified during ATDC5 cell differentiation after intervention with T-2 toxin are presented in Supplementary Table S8.

All differential metabolites identified were mapped to Human Metabolome Database and KEGG Pathway database for pathway analysis to further delineate the underlying functions of the difference metabolites. The main metabolic pathways determined in T-2 intervention group and control groups at each of the time point, such as arachidonic acid metabolism, mitochondrial DNA depletion syndrome, adenine phosphoribosyltransferase deficiency (Supplementary Table S6) and mTOR signaling pathway, inflammatory mediator regulation of TRP channels and thyroid hormone synthesis (Figure 2D–G and Supplementary Table S7).

### 2.3. Integration Analysis of Transcriptomic and Metabolomic

**Figure 3:**
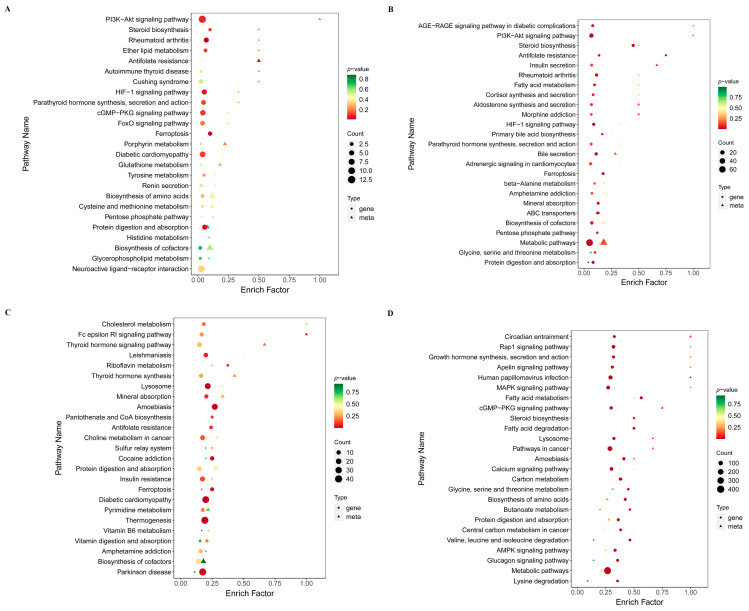
Integration of differential gene expression and metabolite abundance across multiple time points in response to T-2 toxin treatment. (**A**–**D**) Enrichment levels of KEGG pathways (right) showing the degree of enrichment and the number of DEGs and metabolites involved in each pathway at day 3 (**A**), day 7 (**B**), day 14 (**C**), and day 21 (**D**).

At each time point (day 3, 7, 14 and 21), correlation clustering was performed to illustrate the relationships between DEGs and differentially abundant metabolites (Table 1 and Supplementary Table S9). This summary helps identify clusters of genes and metabolites that are coordinately regulated or inversely related in response to T-2 toxin treatment. As described above, many differentially altered transcripts and metabolites enriched in many pathways were identified. For each time point, we displayed the KEGG pathways commonly enriched by DEGs and differentially abundant metabolites (Supplementary Table S10), which provide a comparative view of how many genes and metabolites contribute to each pathway. Additionally, the degree of enrichment and the number of DEGs and metabolites in the enriched KEGG pathways are shown in Supplementary Table S10. The common KEGG map between DEGs and differential metabolites during ATDC5 cell differentiation after intervention with T-2 toxin are shown in Figure 3 and Supplementary Table S10.

### 2.4. Intervention of ATDC5 Cells with T-2 Toxin and BMP Recombinant Protein

**Figure 4:**
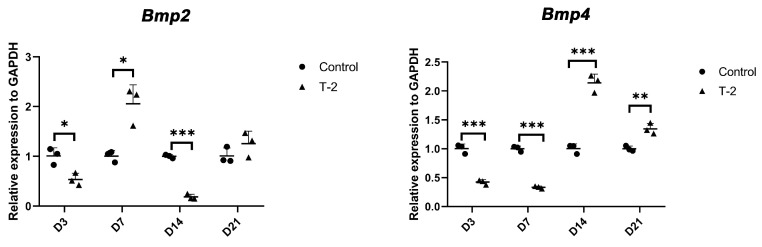
Expression patterns of *Bmp2* and *Bmp4* genes in ATDC5 cells following T-2 toxin and BMP recombinant protein intervention. Statistical significance is indicated as follows: * *p* ≤ 0.05, ** *p* ≤ 0.01, and *** *p* ≤ 0.001.

RT-qPCR were performed to validate the expression patterns of key chondrocyte differentiation-related genes of ATDC5 cells after concurrent intervention with T-2 toxin alone, T-2 toxin combined with BMP2, BMP4, or both BMP2 and BMP4 recombinant proteins during each of the time point. We found that the expression level of *Bmp2* was significantly downregulated on day 3 and day 14, whereas upregulated on day 7 (Figure 4). The expression level of *Bmp4* was significantly downregulated on day 3 and day 7, whereas upregulated on day 14 and day 21 (Figure 4).

**Figure 5:**
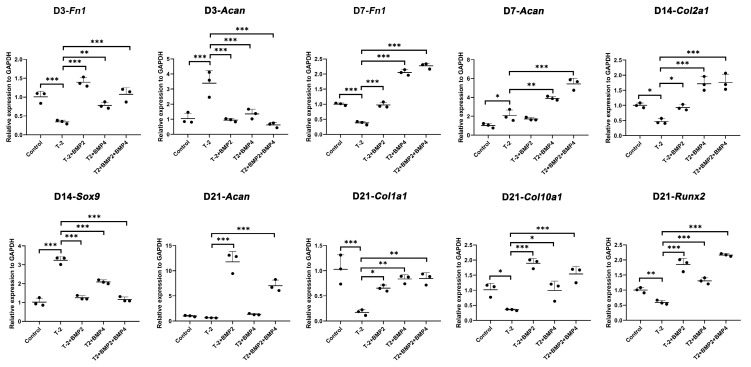
Expression patterns of key chondrocyte differentiation-related genes in ATDC5 cells following T-2 toxin and BMP recombinant protein intervention. Statistical significance is indicated as follows: * *p* ≤ 0.05, ** *p* ≤ 0.01, and *** *p* ≤ 0.001.

The mRNA expression level of *Fn1* were downregulated on day 3 and day 7 by T-2 toxin, which were reversed by concurrent intervention with BMP2, BMP4, or both BMP2 and BMP4 recombinant proteins. While the mRNA expression level of *Acan* were upregulated on day 3 and day 7 by T-2 toxin, which were reversed by concurrent intervention with BMP2, BMP4, or both BMP2 and BMP4 recombinant proteins. *Col2a1* was found to be downregulated by T-2 toxin on day 14, which can be upregulated after concurrent intervention with BMP2, BMP4, or both BMP2 and BMP4 recombinant proteins. *Sox9* was found to be upregulated by T-2 toxin on day 14, which can be downregulated after concurrent intervention with BMP2, BMP4, or both BMP2 and BMP4 recombinant proteins. In addition, we found that the mRNA expression level of *Col1a1*, *Col10a1* and *Runx2* were downregulated by T-2 toxin treatment, while this trend could be reversed by concurrent intervention with BMP2, BMP4, or both BMP2 and BMP4 recombinant proteins. Those results are illustrated in Figure 5.

### 2.5. Alcian Blue Staining Reveals Impaired Extracellular Matrix Formation Under T-2 Toxin Exposure

To further validate the effects of T-2 toxin on chondrocyte matrix synthesis, Alcian blue staining was performed to assess proteoglycan deposition during ATDC5 differentiation (Figure A2). In the control groups, Alcian blue staining intensity increased progressively from day 3 to day 21, consistent with normal maturation and accumulation of cartilage-specific extracellular matrix. In contrast, T-2 toxin-treated cells displayed markedly reduced staining at all time points. The suppression became especially pronounced at the late differentiation stages (D14 and D21), indicating that T-2 toxin disrupts proteoglycan matrix formation and impairs normal chondrocyte maturation.

## 3. Discussion

This study, integrating transcriptomic and metabolomic analyses, identified significant alterations in gene expression and metabolic pathways associated with chondrocyte differentiation in response to T-2 toxin exposure. The findings provide insights into the complex molecular mechanisms underlying the effect of T-2 toxin on chondrocyte differentiation and its potential involvement in the pathogenesis of KBD. Specifically, key genes such as *Col1a1*, *Col2a1*, *Col11a1*, *Smad6*, and *Mmp9*, along with signaling pathways including the BMP signaling pathway, regulation of BMP signaling and collagen-containing extracellular matrix involved in chondrogenesis, were found to be dysregulated, indicating a disruption in the normal process of chondrocyte differentiation.

In this study, several genes involved in chondrocyte differentiation, extracellular matrix organization, and cellular stress responses were found to be significantly up-regulated or down-regulated following T-2 toxin exposure. The up-regulated genes were predominantly enriched in pathways related to oxidative stress, inflammatory signaling, and apoptosis, suggesting that T-2 toxin activates cellular defense mechanisms while simultaneously promoting cell injury. In contrast, the down-regulated genes were primarily associated with cartilage matrix synthesis, cytoskeletal organization, and chondrocyte maturation, indicating an inhibitory effect of T-2 toxin on normal chondrogenic progression. The simultaneous activation of stress-related pathways and suppression of cartilage-specific functional genes highlights a dual mechanism by which T-2 toxin disrupts chondrocyte homeostasis. These molecular alterations are consistent with the pathological features observed in KBD, supporting the hypothesis that T-2 toxin impairs cartilage development by disturbing both protective and structural gene programs.

Previous studies have shown that the coordinated expression of endogenous BMPs, including BMP2 and BMP4, plays a crucial role in the progression of chondrogenic differentiation in ATDC5 cells [20,21]. BMP-2, in particular, stimulates the progression of early-phase differentiation and the formation of calcified matrix, a characteristic end product of terminally differentiated chondrocytes [21]. These findings suggest that BMP-2 can drive both early- and late-phase differentiation in ATDC5 cells [21]. Luyten et al. have also proposed that BMP4 can upregulate the levels of type II collagen and aggrecan mRNAs in cultured articular chondrocytes [22]. A previous study demonstrated that BMP4 expression was significantly reduced in the middle zone of KBD patients compared to normal controls (NC) [23]. Furthermore, BMP2 expression in the deep zone and BMP4 expression in both superficial and deep zones were found to be lower in KBD compared to NC [23]. These results suggest that, as KBD progresses, the expression of BMP2 and BMP4 is diminished [23]. We hypothesize that T-2 toxin, a risk factor for KBD, may exert its effects by targeting BMP2 and BMP4, contributing to the terminal differentiation disorder observed in KBD. Our findings revealed that *Bmp2* expression was significantly downregulated on days 3 and 14, while it was upregulated on day 7. In contrast, *Bmp4* expression was significantly downregulated on days 3 and 7, but upregulated on days 14 and 21. Previous reports have indicated that transcripts for *Bmp2* and *Bmp4* are localized to precartilaginous mesenchymal condensation, and later to the perichondrium [24]. Guo et al. found that the number of viable cells was significantly reduced in the superficial zones of KBD cartilage compared to control cartilage in KBD children, suggesting that T-2 toxin may downregulate *Bmp2* and *Bmp4*, leading to chondrocyte death, necrosis, or apoptosis during the early-phase differentiation of cellular condensation [25].

*Fn1* has been identified as a marker gene for resting chondrocytes [18], functioning as an interactive protein that mediates chondrocyte adhesion [26]. During fracture healing, mesenchymal stem cells are recruited to the fracture site, followed by chondrocyte hypertrophy, a crucial step for endochondral ossification [27,28]. Zhang et al. demonstrated that overexpression of FN1 promoted chondrocyte differentiation and collagen production, leading to the upregulation of *Col2*, *Col1*, and *ColX* during fracture healing [26]. In this study, we observed that T-2 toxin downregulated the mRNA expression of *Fn1* on days 3 and 7, and this effect was reversed by concurrent treatment with BMP2, BMP4, or both BMP2 and BMP4 recombinant proteins. As noted earlier, *Fn1* is a target gene of T-2 toxin, contributing to the pathogenesis of KBD.

The mRNA expression of *Col2a1* was downregulated by T-2 toxin on day 14, while *Col1a1*, *Col10a1*, and *Runx2* were downregulated on day 21. These findings align with previous studies that demonstrated low expression of type II collagen and altered chondrocyte characteristics in KBD. Additionally, reduced type X collagen expression was observed in areas of chondrocyte necrosis in the deep zone of KBD articular cartilage, indicating disturbances in terminal chondrocyte differentiation [29]. Notably, we found that the downregulation of *Col2a1* on day 14 and *Col1a1*, *Col10a1*, and *Runx2* on day 21 could be reversed by concurrent treatment with BMP2, BMP4, or both BMP2 and BMP4 recombinant proteins. Since several BMPs have been shown to offer clinical benefits for treating various conditions, including BMP-2 and BMP-7, which are approved for clinical use in nonunion bone fractures and spinal fusions [30], our findings suggest that recombinant BMP2 and BMP4 proteins may serve as potential candidates for treating early-stage KBD patients.

Beyond the BMP signaling cascade, our transcriptomic data revealed that T-2 toxin exposure also perturbed several other pathways known to regulate chondrocyte differentiation, including the Wnt [31], TGF-β [32], and FGF signaling pathways. Genes such as *Wnt9a*, *Tgfbr31*, and *Fgfr4* showed significant alterations, suggesting that T-2 toxin may cause a broader disruption of chondrogenic regulatory networks. Crosstalk among these pathways is well recognized in cartilage biology—BMP and TGF-β signaling cooperatively promote extracellular matrix synthesis, while Wnt and FGF pathways are involved in balancing chondrocyte proliferation and hypertrophy. The coordinated dysregulation of these pathways may therefore amplify the impairment of endochondral ossification observed following T-2 toxin exposure. Together, these results highlight that the effects of T-2 toxin are not limited to the BMP axis but extend to multiple interconnected signaling networks governing cartilage homeostasis. This broader molecular disturbance may underlie the complex pathological manifestations of cartilage degeneration seen in KBD.

In addition to disrupting chondrocyte differentiation, our transcriptomic data suggest that T-2 toxin may induce apoptotic activation, which could further exacerbate cartilage degeneration. Several pro-apoptotic genes, including *BAX*, *CASP3*, *CASP9*, and *BCL2*, exhibited upregulated expression following toxin exposure, indicating engagement of both the intrinsic mitochondrial and p53-dependent apoptotic pathways. These molecular changes align with previous reports showing that T-2 toxin can trigger apoptosis through excessive reactive oxygen species (ROS) production, mitochondrial dysfunction, and endoplasmic reticulum stress in various cell types, including chondrocytes [33,34]. The concomitant downregulation of differentiation markers such as *COL2A1* and *SOX9* further supports a model in which apoptosis and impaired differentiation act synergistically to compromise cartilage matrix formation. Although we did not directly quantify apoptotic activity in this study, these transcriptomic signatures, together with prior mechanistic evidence, strongly imply that T-2 toxin-induced apoptosis may represent a key downstream event linking toxic exposure to cartilage injury. Future work employing flow cytometry, TUNEL staining, or caspase activity assays will be essential to experimentally validate this hypothesis.

By integrating transcriptomic and metabolomic datasets, this study reveals coherent molecular alterations that collectively explain how T-2 toxin disrupts chondrocyte differentiation. The suppression of *Bmp2* and *Bmp4* expression at early and mid-differentiation stages was accompanied by metabolic disturbances in lipid-related pathways, particularly arachidonic acid metabolism, which is known to modulate BMP activity through inflammation-associated lipid mediators [35,36]. These results imply that T-2 toxin may impair the BMP signaling axis both transcriptionally and metabolically, creating a feed-forward loop that exacerbates chondrocyte dysfunction. In addition, joint enrichment of DEGs and metabolites in oxidative stress and ferroptosis-related pathways further supports a multi-layered disruption of cellular homeostasis. The correlation highlights clusters of genes and metabolites that respond in a coordinated manner to T-2 exposure, emphasizing that metabolic reprogramming is tightly linked to transcriptional regulation. Together, these integrated findings provide a unified mechanistic framework demonstrating that T-2 toxin induces coupled transcriptional and metabolic dysregulation, ultimately impairing chondrocyte differentiation and contributing to KBD-related cartilage pathology.

Despite the strengths of our multi-omics approach, several limitations should be acknowledged. First, although we evaluated the rescue effects of BMP2 and BMP4 on T-2 toxin-induced dysregulation of chondrocyte differentiation, BMP-only groups were not included in the initial experimental design. As a result, we were unable to distinguish the direct transcriptional or metabolic effects of BMP supplementation from those specifically counteracting T-2 toxin exposure. Future studies incorporating BMP-only controls will be essential to more clearly delineate the intrinsic molecular actions of BMP signaling in chondrocyte development. Second, the concentration of 8 ng/mL T-2 toxin used in this work was selected based on preliminary cytotoxicity screening, but a full dose–response analysis was not performed. In addition, the fixed 48 h exposure reflects only short-term toxicity and does not fully capture the chronic, cumulative toxic effects relevant to the pathogenesis of KBD. To address this limitation, future investigations will incorporate long-term exposure models and in vivo experiments to better characterize dose-dependent responses and the sustained impact of T-2 toxin and BMP signaling on cartilage homeostasis. While Alcian blue staining has been included to visualize matrix deposition, we acknowledge that protein-level validation was not performed in the current work. Future studies will expand to Western blotting, immunofluorescence, and additional histological assessments to further confirm BMP signaling activity and matrix formation at the protein level.

## 4. Materials and Methods

### 4.1. Cell Viability Assay and Sample Preparation

The ATDC5 cells (BLUEFBIO™) were then induced with ITS (41400045, Thermo, Rochester, NY, USA) for 3, 7, 14, and 21 days to simulate the early stage of chondrocyte differentiation, proliferation stage, and hypertrophic calcification stage, respectively. Cell viability was measured using the cell counting kit-8 (CCK8, NCM Biotech, Suzhou, China) according to the manufacturer’s instructions. In brief, chondrocytes were seeded in the 96 well-plate (5000 cells/well) and incubated in DMEM/F12 (HyClone, Logan, UT, USA) with 10% fetal bovine serum (Sijiqing, Zhejiang Tianhang Biotechnology Company, Hangzhou, China), 1% penicillin and streptomycin (HyClone, Logan, UT, USA) at 37 °C for 24 h. T-2 toxin (MSS1023, Pribolab, Qingdao, China) was first dissolved in dimethyl sulfoxide (DMSO, Sigma-Aldrich, St. Louis, MO, USA) to prepare a stock solution and subsequently diluted in culture medium to a final working concentration. The final concentration of DMSO in all T-2 toxin-treated groups was maintained below 0.1% (*v*/*v*). A solvent control (vehicle control) group was included in all experiments, in which cells were treated with culture medium containing an equivalent concentration of DMSO (<0.1% *v*/*v*) but without T-2 toxin, to ensure that any observed effects were attributable solely to T-2 toxin exposure rather than to the solvent itself.

**Figure A1:**
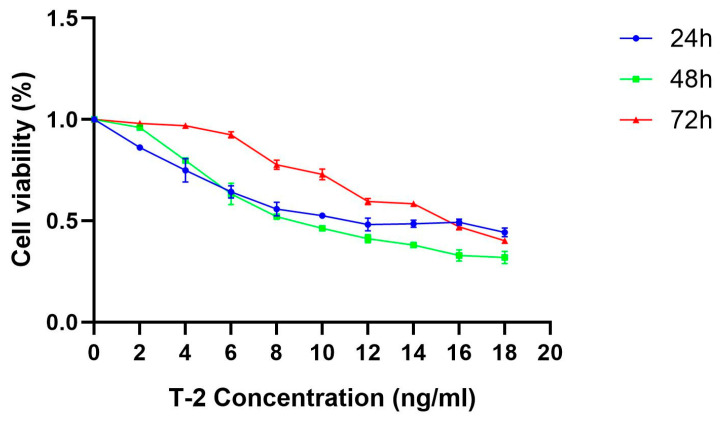
The cytotoxicity of T-2 toxin on ATDC5 cell viability at 24 h, 48 h and 72 h, respectively.

**Figure A2:**
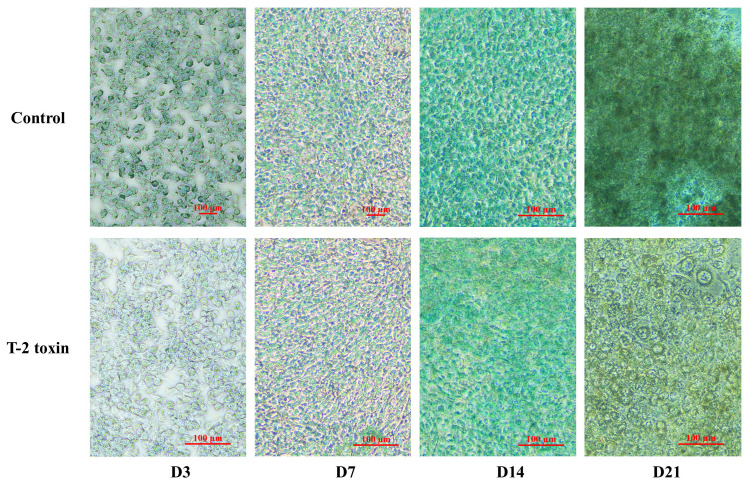
Alcian blue staining of ATDC5 cells at days 3, 7, 14, and 21 of chondrogenic differentiation under normal conditions and T-2 toxin treatment (8 ng/mL). Cells were fixed and stained with 1% Alcian blue (pH 2.5) to assess proteoglycan matrix deposition. Representative images were captured using an inverted phase-contrast microscope (Panasonic, Kadoma, Japan). Compared with the control group, T-2 toxin-exposed cells exhibited visibly reduced Alcian blue staining intensity across all differentiation stages, indicating impaired extracellular matrix synthesis and disrupted chondrogenic differentiation.

The chondrocytes were then treated with different concentrations of T-2 toxin for 24 h, 48 h and 72 h, respectively. Afterwards, 10 μL CCK-8 solution was added to each well. The absorbance of each well at 450 nm was measured by a microplate reader (Model 3550, Bio-Rad, Hercules, CA, USA) after the 2 h incubation period at 37 °C. The cytotoxicity of T-2 toxin on ATDC5 chondrocytes viability were evaluated by CCK-8 assay at 24 h, 48 h and 72 h, respectively. As depicted in Figure A1, the cell viability was decreased with the increase in T-2 toxin concentration at 24 h, 48 h and 72 h, respectively. Notably, it was observed that T-2 toxin exhibited obvious toxicity on chondrocytes (about 50% cell viability) at concentrations exceeding 8 ng/mL. Hence, the concentrations of 8 ng/mL at 48 h were used for the subsequent in vitro experiments.

For transcriptomic sequencing, ATDC5 cells were cultured with ITS [37] to induce differentiation for 3, 7, 14, and 21 days. At each time point, the cells were exposed to T-2 toxin (8 ng/mL) for 48 h. Following the treatment, 1 mL of TRIzol reagent (Invitrogen, Waltham, MA, USA) was added, and the cells were lysed by repeated pipetting. The resulting lysates were immediately snap-frozen in liquid nitrogen for subsequent RNA extraction. For metabolomic sequencing, the experimental conditions mirrored those used for transcriptomic analysis. After the 48 h T-2 toxin treatment, 1 mL of PBS was added to the cells, followed by gentle pipetting. The cell suspensions were centrifuged at 4 °C to pellet the cells, and the supernatant was discarded. The cell pellets were promptly frozen in liquid nitrogen for further metabolomic analysis.

### 4.2. RNA Sequencing Analysis

Each sample, prepared in triplicate, was submitted for mRNA sequencing. The construction of cDNA libraries and subsequent sequencing were conducted using the Illumina platform (Illumina HiSeq 4000, Inc., San Diego, CA, USA) by Metware Biotechnology Co., Ltd. (Wuhan, China). Differential gene expression analysis between groups was carried out using DESeq2 software (v1.22.1), while pairwise comparisons between individual samples were performed using edgeR (v3.24.3) [38,39]. Genes with a false discovery rate (FDR) of less than 0.05 and an absolute fold change in ≥2 were considered differentially expressed [40]. These differentially expressed genes (DEGs) were subsequently analyzed for functional enrichment using Gene Ontology (GO) and Kyoto Encyclopedia of Genes and Genomes (KEGG) pathway enrichment analyses [41,42].

### 4.3. Metabolomic Analysis

Metabolomics profiling of the samples was performed using an LC-ESI-MS/MS system (UPLC, ExionLC AD, https://sciex.com.cn/, Shanghai, China; MS, QTRAP^®^ System, https://sciex.com/, Marlborough, MA, USA). The column temperature was maintained at 40 °C. Unsupervised principal component analysis (PCA) was performed using the prcomp (v3.5.1) function in R (v3.5.1) (www.r-project.org) after unit variance scaling of the data. For two-group comparisons, differential metabolites were identified based on Variable Importance in Projection (VIP ≥ 1) and an absolute Log2 fold change (|Log2FC| ≥ 1.0). Before orthogonal partial least squares discriminant analysis (OPLS-DA), the data were log-transformed (log2) and mean-centered. To prevent overfitting, a permutation test with 200 permutations was conducted. Identified metabolites were annotated using the KEGG Compound database (http://www.kegg.jp/kegg/compound/ (accessed on 11 December 2024)) and subsequently mapped to pathways in the KEGG Pathway database (http://www.kegg.jp/kegg/pathway.html (accessed on 11 December 2024)). Pathways with significantly regulated metabolites were further subjected to metabolite set enrichment analysis (MSEA) using the Human Metabolome Database (HMDB). The significance of the enrichment was determined using hypergeometric test *p*-values.

### 4.4. Integrated Analysis of Transcriptomic and Metabolomic Data

To systematically and comprehensively understand the molecular changes in ATDC5 cells under T-2 toxin exposure at different time points, transcriptome and metabolome data were integrated. For each time point, we identified the KEGG pathways that were commonly enriched by DEGs and differentially abundant metabolites. It highlights the biological processes significantly affected by T-2 toxin exposure. This integrated analysis offers a comprehensive view, enabling a direct comparison of enrichment levels across different omics layers and identifying key pathways implicated in the response to T-2 toxin.

### 4.5. Intervention of ATDC5 Cells with T-2 Toxin and BMP Recombinant Protein

Previous studies have shown that the coordinated expression of endogenous BMPs, such as BMP2 and BMP4, plays a key role in the progression of chondrogenic differentiation in ATDC5 cells [20,21]. Transcriptomic analysis revealed a significant downregulation of BMP4 during chondrocyte differentiation following T-2 toxin exposure. As essential components of the BMP signaling pathway, these proteins are critical in regulating cartilage development and endochondral ossification. Therefore, they were selected for further investigation to assess their potential involvement in the T-2 toxin-induced disruption of chondrocyte differentiation. In brief, ATDC5 cells were induced to differentiate at various time points (days 3, 7, 14, and 21) with exposure to T-2 toxin alone, or in combination with BMP2 (HY-P7006, MedChemExpress, Monmouth Junction, NJ, USA), BMP4 (HY-P74379, MedChemExpress), or both recombinant proteins. BMP2 and BMP4 were applied at a final concentration of 100 ng/mL and were administered concurrently with T-2 toxin treatment, remaining in the culture medium throughout the 48 h exposure period. Real-time quantitative polymerase chain reaction (RT-qPCR) was employed to measure the expression levels of chondrocyte differentiation-related genes (*Fn1*, *Sox9*, *Runx2*, *Acan*, *Col1a1*, *Col2a1*, and *Col10a1*) in each treatment group.

### 4.6. Quantitative Real-Time Polymerase Chain Reaction Analysis (RT-qPCR)

Total RNA was isolated from the cultured cells using TRIzol reagent (Thermo Fisher Scientific Inc., Waltham, MA, USA) and subsequently reverse transcribed into cDNA using the 5X Evo M-MLV RT Master Mix (AG, Wuhan, China), following the manufacturer’s instructions. Real-time PCR analysis was performed with SYBR Green Pro Taq HS (AG, Wuhan, China) on a 7500 Real-Time PCR System (Applied Biosystems, Waltham, MA, USA). The relative mRNA expression levels were calculated using the delta-delta Ct method.

### 4.7. Alcian Blue Staining

Alcian blue staining was performed to evaluate proteoglycan deposition during chondrocyte differentiation. ATDC5 cells were cultured and induced to differentiate as described above. At the indicated time points (days 3, 7, 14, and 21), cells from both the control and T-2 toxin-treated groups were gently washed twice with PBS (HyClone, Logan, UT, USA) and fixed with 4% paraformaldehyde (PFA, Biosharp, Hefei, China) for 15 min at room temperature. After fixation, cells were rinsed with distilled water and incubated with 1% Alcian Blue Staining Kit (pH 2.5, Beyotime, Shanghai, China) for 30 min. Following staining, cells were rinsed with distilled water for three times. Stained cultures were imaged using an inverted light microscope (Panasonic, Kadoma, Japan), and proteoglycan-rich extracellular matrix was visually assessed based on the intensity and distribution of the blue coloration.

### 4.8. Statistical Analysis

All data are expressed as mean ± standard deviation (SD), with statistical analyses performed using GraphPad Prism 8.3.0 and SPSS 21.0. Dunnett’s *t*-test was applied for data with homogeneity of variance, and Dunnett’s T test was used for data with heterogeneity of variance. Comparisons between multiple groups were conducted using one-way ANOVA. For data with non-normal distributions, non-parametric tests were applied, and a *p*-value of <0.05 was considered statistically significant.

## 5. Conclusions

In conclusion, our study provides a comprehensive analysis of the effects of T-2 toxin exposure on chondrocyte differentiation and its involvement in the pathogenesis of KBD, with a focus on BMP signaling. Through transcriptomic and metabolomic analyses, we identified significant changes in gene expression and metabolic pathways associated with chondrocyte differentiation upon T-2 toxin exposure. Validation studies confirmed the dysregulation of key chondrogenic genes and revealed altered BMP expression during the differentiation process of ATDC5 cells. Additionally, combination treatment experiments highlighted the intricate interactions between T-2 toxin and BMPs, suggesting potential therapeutic targets for KBD intervention. Specifically, we emphasize that future work should validate the major transcriptomic and metabolomic alterations—particularly the disruption of BMP signaling—in primary human chondrocytes, organoid systems, or in vivo chronic-exposure models that better reflect the pathological context of KBD. We also highlight the therapeutic potential of targeting the BMP signaling pathway, recommending further investigation into BMP agonists, recombinant BMP proteins, or small-molecule modulators as possible interventions to mitigate T-2 toxin-induced cartilage damage. In addition, we propose integrating proteomics, epigenetic profiling, and functional assays of cartilage matrix deposition and morphology to achieve a more comprehensive mechanistic understanding and support the development of effective therapeutic strategies. Overall, our findings deepen the understanding of the molecular mechanisms underlying KBD and offer insights into possible strategies for the development of targeted therapies aimed at alleviating the burden of this debilitating disease.

## Figures and Tables

**Table 1:** Integrated transcriptomic and metabolomic pathways commonly altered by T-2 toxin across chondrocyte differentiation stages.

Group	KEGG Pathway	Description	Biological Relevance
D3_C vs. D3_T2	ko04216	Ferroptosis	Early activation of ferroptosis suggests that T-2 toxin triggers oxidative stress and disrupts redox homeostasis at the initial stage of chondrogenic differentiation, potentially impairing BMP–mediated survival and lineage commitment.
D3_C vs. D3_T2	ko05323	Rheumatoid arthritis	Enrichment of RA-related pathways reflects early induction of inflammation and catabolic activity by T-2 toxin, consistent with ECM degradation and reduced expression of chondrogenic markers.
D7_C vs. D7_T2	ko01100	Steroid biosynthesis	Disruption of steroid biosynthesis may alter membrane composition and intracellular signaling required for chondrocyte proliferation and differentiation, interfering with TGF-β/BMP-dependent pathways.
D7_C vs. D7_T2	ko04978	Mineral absorption	Impaired ion homeostasis affects collagen synthesis and enzymatic activities essential for cartilage ECM assembly, compromising the progression of normal chondrogenesis.
D14_C vs. D14_T2	ko04142	Lysosome	Lysosomal pathway activation indicates enhanced cellular stress, contributing to ECM breakdown and reduced matrix accumulation during the hypertrophic transition phase.
D14_C vs. D14_T2	ko04979	Cholesterol metabolism	Altered cholesterol homeostasis may impair chondrocyte membrane signaling and metabolic adaptation, weakening anabolic processes required for cartilage maturation.
D21_C vs. D21_T2	ko00480	Metabolic pathways	Widespread metabolic disruption suggests cumulative toxic effects of T-2 toxin on late-stage cartilage formation, contributing to impaired ECM deposition and skeletal tissue integrity.
D21_C vs. D21_T2	ko01212	Fatty acid metabolism	Dysregulated lipid metabolism affects energy production and chondrocyte maturation, potentially hindering BMP-7–linked anabolic signaling and matrix synthesis.

## Data Availability

The data presented in this study are available upon request from the corresponding author (fzhxjtu@xjtu.edu.cn) due to privacy.

## Supplementary Materials

The following supporting information can be downloaded at https://www.mdpi.com/article/10.3390/ijms262411858/s1.

## Abbreviations

The following abbreviations are used in this manuscript:

KBD	Kashin–Beck disease
ITS	Insulin, Transferrin, Selenium
CCK8	cell counting kit-8
DMSO	dimethyl sulfoxide
FDR	false discovery rate
DEGs	differentially expressed genes
GO	Gene Ontology
KEGG	Kyoto Encyclopedia of Genes and Genomes
LC-ESI-MS/MS	liquid chromatography-electrospray ionization-tandem mass spectrometry
PCA	principal component analysis
VIP	Variable Importance in Projection
FC	fold change
OPLS-DA	orthogonal partial least squares discriminant analysis
MSEA	metabolite set enrichment analysis
HMDB	Human Metabolome Database
RT-qPCR	Real-time quantitative polymerase chain reaction
PFA	paraformaldehyde
SD	standard deviation
NC	normal controls
